# Address

**Published:** 1866-01

**Authors:** Geo. L. Paine


### ADDRESS.

BY DR. GEO. L. PAINE.

**Read before the Mad River Valley Dental Association.**

"To save is better than to destroy." The indestructibility of matter is an axiomatic principle of philosophy. Nothing is ever wasted in Nature's great laboratory; but everything is turned to practical account. True, new forms are assumed, and new combinations arranged — yet, *not* a particle of matter is ever lost, The leaf performs its functions in its season — then withers — dies — drops to earth and turns to dust — each particle thereof, though lost as a leaf, is *not*, therefore, lost as to its ultimate particles, which are imperishable. So of its parent, the tree. So, of all material things. While changes are going on incessantly in the laboratory of nature, there is no destruction of matter: it is eternal, as is its Maker, God. While we are taught on all sides that nature is a great conservator, let us imitate her and try to be conservative, as a profession, rather than destructive Our art, unlike sculpture or painting, which genius impresses on marble and canvas, and is to some extent imperishable—has to deal with perishable materials, and must therefore, as to real benefits be temporary; hedged about and hampered as it is by decay and mortality. Yet, dental art should be more conservative than it has been—than it is—than we fear it will be. Higher skill, broader culture and conscience must have a more lively existence among the dental profession; then the elysian period shall have been reached; our patients will be so taught to educate their children as to secure for them sound dentures, and to keep them so, through life. In this golden age—if attained—the forceps will rest in idleness, instead of being kept bright with active service; the vulcanizer, the great center of attraction with most dentists, will be regarded as an heirloom of a past generation. The preservation, not the extraction of a tooth, will then, as it should now, enlist all the energies of the devoted and conscientious dentist, who, with the co-operation of his patient, whom he has well trained, will be able to succeed in saving from the rapacious jaws of the bloody forceps.

When a community are taught, by whatever means may be employed; whether by tracts, or brief essays published in the daily or weekly papers; how to live; how to take care of their pearls of great price; if any member of the dental circle is on the sick-list, from any cause; for instance, from a wound received in an engagement with a refractory particle of matter in the food, which it is his allotted duty to grind, or, from a cold, caught because of a deficiency in his outward apparel; his health will be looked after with as much solicitude, as though the body corporate itself was the subject of disease.

When we are sick, we employ the most skillful and experienced talent—the most careful nurses, and leave no means untried, to aid nature in throwing off the disease. So our people should be taught to act with regard to their teeth—not to take them to some itinerant mountebank, or tooth assassin, whose only cure is extraction — but the ailing organ should be submitted to the care of an experienced and enlightened and conscientious dentist, who labors, *primarily*, for the good of his patients; and *secondarily*, for himself. The province of such an operator is to *save*, and he will succeed, other things being equal, having the hearty co-operation of his patients, whom he has duly trained. His patients are not only amply compensated for the fee, however liberal, which he charges for work *faithfully* done, but for the advice which he gives, which, when followed, is above all price. This sketch, we admit, borders more on *ideality* than *reality*, nevertheless, it is such as we should daily study and adopt to the extent of our ability.

We must elevate the standard of our professional education and conduct, and demand higher and still higher qualifications of those who are practicing, or preparing to practice our specialty. We require stimulating and guarding *constantly* — else we retrograde. We need to be often reminded of our duty to our patients and to each other — else we neglect it. We must become students — otherwise we remain mere mechanical dentists. We must study, or we make no progress — and then will be left behind by our more ambitious brethren. We either advance, or fall back. The world moves! — so must we, or be left alone to fossilize and rust from torpidity and inaction. Where would the lawyer land, if he neglected to keep pace with his brethren at the bar? Where the physician? Where the theologian, and where the teacher? Failure would be the result! They would all go to the tomb of the capulets, " unwept, unhonored and unsung." "There is no royal road to Geometry"— the way is hedged about with difficulties and obstacles which can only be surmounted by patient toil and study. Books, lectures, essays and studies, are the finger boards, pointing out the only safe way; if *these* are ignored, we are liable to miss the way — go astray, and get lost in the wilderness of ignorance. We therefore, exhort you all, my brethren, to buckle on your armor and determine to accomplish something noble for yourselves and the profession. That you may be live men and not drones in the dental hive. When we are thus actuated, we may reasonably expect that our profession will stand side by side with the other learned professions. Nothing but solid attainments can elevate us. Mere skill, as operatives, however praiseworthy, may give us a local name and fame, but nothing more. There must be a union of science and skill to produce immortal results.

What has elevated, ennobled and dignified the medical profession, and given it so much power and influence? Science. Who occupy the highest and most lucrative positions, both in the medical and dental professions? Those who read, study, write and think the most. Who wield the most influence in the learned professions? Those best educated and most studious.

Let us, for a few moments be permitted to take a bird's eye view of our profession — the qualifications, standing and deportment of many of its members. Have a majority of those who are wielding the forceps and excavator passed through that curriculum of study and training, at Dental Colleges for instance, which qualifies them for the proper discharge of professional duties? About 10 in 500, the statistics say. This is about the proportion. How many have ever read and digested the text books of our specialty? — to say nothing about Anatomy, Physiology, Pathology, Surgery, Chemistry, &c. How many dentists *among us*, own, and have read and digested the volumes of our periodical literature? Few, we apprehend. We are sorry to say it — but truth demands it at our hands, that many dentists, *among us*, are not much given to reading — not much devoted to study — and less to writing. The information that many possess, touching the great principles that underlie our profession, is meagre in the extreme — such as may be picked up in any office by any ordinary observer. For instance they know, some of them — that incisors have one root — superior molars, three —inferior molars, two — that a cavity of decay, provided the nerve is not exposed, must be cleansed of carious matter previous to filling, and so shaped as to retain the same — that an aching tooth must be extracted and that the chief end of the dentist is to make artificial teeth. The ambition of men of this ilk, take an exclusively mechanical turn, and runs wholly in the direction of artificial teeth — albeit they announce themselves as M. D's and Surgeon Dentists. Their forte is by much nice talk, and a good deal of "soft solder" to persuade their credulous patients to have their teeth extracted, so they can insert new ones — a much easier task for them than to insert good *gold plugs* in the natural teeth, whereby they might be saved. Their advice is taken, and their pockets are filled with greenbacks, and their patients mouths with rubber and dental crockery. Thus is our noble profession demoralized—prostituted to bare ends by the demon of quackery, which flourishes most in him whose brain is poverty stricken, and whose soul is as barren as the sands of the desert. What do these fellows do for the good of humanity? What care they for humanity? Do they try to preserve God's beautiful creations? Are they not rather vandals? — entering with defiled hands and bloody garments the "holy of holies" — despoiling and overthrowing the beautiful works. Creative conservative dentistry is not their end or ambition. Their dentistry is radical. They prefer not to bother their heads with the laws of Physiology, and Pathology — they ignore nerves and abscesses — with them, the fate of a tooth is sealed when it aches — or periostitis is developed. The rules of science is to them a sealed book. They require about as much of it as the cooper or shoemaker. They pride themselves on what nature has done for them; as we heard one of them say once, when asked if he had attended a course of lectures in a dental college. He has since quit the business, having failed to satisfy the public, and make a decent living and gone to preaching, in which vocation he succeeds much better. These men are mere copyists, doing parrot like, whatever they undertake; and whatever of good they may be guilty of doing, is due to the seed sown by some one, who was not too lazy to study—who was not too wise in his own conceit, to learn — who burrowed into the mine of knowledge and appropriated everything that could possibly aid him, in his efforts to aid, and ameliorate the condition of his fellow men. Such a man feels prouder of a victory achieved over a rebellious tooth, which he has saved from destruction and the forceps — than of a finely finished and well adapted set of teeth. The former is a triumph of science and high skill; a feat that puts him on an equality with physicians and surgeons, and justly entitles him to the appellation of "Dental Surgeon;" while the other is simply a piece of mechanism, demanding no higher skill than that employed by the tailors, whose beau ideal is "perfect fits."

But little learning is required, now-a-days — when gold is out of fashion, as well as the workers thereof — to make a set of teeth, when vulcanite work is all the rage — when sets of teeth, as the posters innumerable inform us, as well as the pine shingles tacked to telegraph poles and gate posts all over the country advise us, can be made by the aid of steam and gas for Ten Dollars! Secundem artem—Blessed Era. People can now get their teeth extracted by lightning and made by steam—all for Ten Dollars. Who would *not* have his teeth all pulled out? To set up in business, only a three months' apprenticeship, a vulcanizer—a box of rubber and a grindstone and plenty of gas are necessary Then if patients don't come in they go for them. May God save us from the doom, degradation and disgrace that surely await us, as a profession if this mode of business obtain in this land—let us expose the quackery and ignorance of those men, whose breath is poison, whose touch is death! Gentlemen, we say it in sorrow that the status of many of the profession in *our* valley is low, vile and disgraceful, and should be improved, elevated. There are in our circuit some 50 or 60 dentists, in a neighboring city there are some 16 in active practice; here 6 or 7, of these how many are here—how many *ever* attend any dental meetings, or manifest any sort of interest in the progress or welfare of our calling. We must work a revolution in this matter, and create a public sentiment that will drive these men here or drive them out. While we are, out of respect to Truth, obliged to make such a humiliating statement respecting the status of dentists within our bounds, we are glad to affirm that there are suns and stars in our dental firmament, with noble families of satellites revolving around them—men who would honor any of the learned professions by their scientific attainments—who would elevate any calling by their skill, and adorn any social circle or community by their manly bearing, honorable conduct and gentlemanly deportment.

The normal and conservative practice of dental surgery is a noble vocation, which numbers in its ranks men who stand high for their skill and extensive and varied attainments. Their work will live after them not only in the dentures of their grateful patients, but in the pages of our professional literature.

Let us be conservators of our profession. Let us determine that our practice shall be normal—that we *will try* to do good and not evil, *knowing* by that we will try to save instead of destroy. Possibly we may not have so much money when we are ordered to ground arms; but we shall hope to have, instead, *clean money* and a conscience void of offense, both in the sight of God and man.

How many of us are purged of the filth and dross of an irregular radical practice? How many of us are so thoroughly grounded in the faith of an honorable code, that we would suffer want rather than do wrong to a patient or dental brother.

